# A Next-Generation Sequencing Study in a Cohort of Sicilian Patients with Parkinson’s Disease

**DOI:** 10.3390/biomedicines11123118

**Published:** 2023-11-22

**Authors:** Michele Salemi, Giuseppe Lanza, Maria Grazia Salluzzo, Francesca A. Schillaci, Francesco Domenico Di Blasi, Angela Cordella, Salvatore Caniglia, Bartolo Lanuzza, Manuela Morreale, Pietro Marano, Mariangela Tripodi, Raffaele Ferri

**Affiliations:** 1 Oasi Research Institute—IRCCS, 94018 Troina, EN, Italy; msalemi@oasi.en.it (M.S.); msalluzzo@oasi.en.it (M.G.S.); fran7.sch@gmail.com (F.A.S.); fdiblasi@oasi.en.it (F.D.D.B.); scaniglia@oasi.en.it (S.C.); blanuzza@oasi.en.it (B.L.); mmorreale@oasi.en.it (M.M.); pmarano@oasi.en.it (P.M.); mtripodi@oasi.en.it (M.T.); rferri@oasi.en.it (R.F.); 2 Department of Surgery and Medical-Surgical Specialties, University of Catania, 95123 Catania, CT, Italy; 3 Genomix4Life Srl, 84081 Baronissi, SA, Italy; angela.cordella@genomix4life.com; 4 Genome Research Center for Health—CRGS, 84081 Baronissi, SA, Italy

**Keywords:** Parkinson’s disease, NGS, movement disorders, gene variants

## Abstract

Parkinson’s disease (PD) is a multisystem and multifactorial disorder and, therefore, the application of modern genetic techniques may assist in unraveling its complex pathophysiology. We conducted a clinical–demographic evaluation of 126 patients with PD, all of whom were Caucasian and of Sicilian ancestry. DNA was extracted from the peripheral blood for each patient, followed by sequencing using a Next-Generation Sequencing system. This system was based on a custom gene panel comprising 162 genes. The sample underwent further filtering, taking into account the allele frequencies of genetic variants, their presence in the Human Gene Mutation Database, and their association in the literature with PD or other movement/neurodegenerative disorders. The largest number of variants was identified in the leucine-rich repeat kinase 2 (*LRRK2*) gene. However, variants in other genes, such as acid beta-glucosidase (*GBA*), DNA polymerase gamma catalytic subunit (*POLG*), and parkin RBR E3 ubiquitin protein ligase (*PRKN*), were also discovered. Interestingly, some of these variants had not been previously associated with PD. Enhancing our understanding of the genetic basis of PD and identifying new variants possibly linked to the disease will contribute to improved diagnostic accuracy, therapeutic developments, and prognostic insights for affected individuals.

## 1. Introduction

Parkinson’s disease (PD) is the most common neurodegenerative disease worldwide after Alzheimer’s dementia (AD) and is the foremost degenerative movement disorder. Pathophysiologically, PD is a complex disorder [1,2] characterized by a heterogeneous clinical presentation that includes both motor symptoms, which remain the gold standard for diagnosis, and non-motor symptoms, which are, however, equally prevalent and disabling [3,4]. The primary motor symptoms encompass bradykinesia, resting tremor, muscular rigidity, and, later in the disease course, balance disturbances. Meanwhile, non-motor signs include late-life depression, cognitive decline, rapid eye movement sleep behavior disorder (RBD), hyposmia, and constipation, among others [2,5]. Notably, non-motor symptoms can precede motor manifestations by several years, defining the so-called prodromal phase of PD [2,6,7]. Accordingly, PD can be divided into three stages: preclinical PD, in which neurodegeneration has begun but no clinical signs or symptoms are yet evident; pre-motor or prodromal PD, where clinical signs and/or symptoms are present but they are still insufficient for a PD diagnosis; and clinical PD, where the diagnostic criteria are fully met [6]. The main pathophysiological hallmark of PD is the presence of Lewy Bodies, i.e., intracytoplasmic aggregates of insoluble alpha-synuclein, and the loss of dopaminergic neurons, especially within the midbrain substantia nigra, pars compacta [2].

PD is currently diagnosed through a neurological examination and medical history, as there are no laboratory tests or instrumental exams available for a definitive diagnosis. However, PD is a pleomorphic disease, with the presence of both motor and non-motor symptoms confirming its pluri-systemic and multifactorial nature [2,5], influenced by various genetic, neurobiological, and environmental factors working synergistically [4,8]. Therefore, understanding the molecular mechanisms underlying PD is crucial for both clinicians and researchers. In this context, the identification of the genetic basis of the disease would greatly aid physicians in improving diagnoses and developing new drugs [9,10]. In major neurodegenerative diseases, such as PD, AD, amyotrophic lateral sclerosis (ALS), and frontotemporal dementia, molecular approaches have recently led to the identification of numerous genetic mutations that significantly affect the disease development, onset, and progression [5,9]. One of the first mutated genes identified in PD patients was the alpha-synuclein (*SNCA*) gene, encoding for the alpha-synuclein protein. This was followed by the discovery of mutations in other relevant genes, such as leucine-rich repeat kinase 2 (*LRRK2*), vacuolar protein sorting 35 (*VPS35*), PTEN-induced kinase 1 (*PINK1*), ATPase 13A2 (*ATP13A2*), phospholipase A2 group VI (*PLA2G6*), and acid beta-glucosidase (*GBA*). However, over the years, several other loci have been highlighted, some of which are associated with peculiar phenotypes [3,5].

Given these considerations, detecting new mutations in neurodegenerative diseases is of paramount importance in disentangling the complex genomic and clinical manifestations of PD, ultimately paving the way for innovative disease-modifying treatments. In this context, the Next-Generation Sequencing (NGS) techniques have emerged as a modern approach, enabling the examination of millions of sequences simultaneously. Consequently, the once-unknown causes of rare molecular diagnoses can be now determined relatively quickly and with a high accuracy and reliability. However, it Is essential to handle the vast amount of generated data with caution, particularly in patient diagnosis and management, since everyone possesses a unique genome. Nevertheless, NGS has revolutionized conventional diagnostic and therapeutic strategies, transitioning them into modern sequencing and individual genomic mapping [11].

Population-based studies have revealed that approximately 5–10% of PD patients have a genetic form of the disease. Traditionally, PD has been associated with at least 13 loci and 9 genes, including autosomal dominant forms such as Parkinson disease 1 (*PARK1*) and *SNCA/PARK4*, ubiquitin C-terminal hydrolase L1 (*UCHL1/PARK5; PARK8/LRRK2*; GRB10 interacting GYF protein 2 (*GIGYF2/PARK11*), and HtrA serine peptidase 2 (*HTRA2/PARK13/OMI*), as well as autosomal recessive forms like *PRKN/PARK2/Parkin; PARK6/PINK1;* and *PARK7/DJ-1/PARK9/ATP13A2* [12]. In recent years, our understanding of PD genetics has advanced significantly, with the identification of five additional genes causing monogenic forms [13] and the recognition of 11 loci as risk modifiers for common forms of PD [14].

More recently, two independent studies utilizing Whole Exome Sequencing (WES) in Austrian and Swiss kindreds detected the same p.D620N mutation (c.1858G>A) in the *VPS35* gene as a causative of autosomal dominant PD [15,16]. As known, *VPS35* plays a role in retrograde transport from endosomes to the trans-Golgi network and, as a consequence, the p.D620N mutation may cause a dysfunctional endosomal–lysosomal trafficking due to impaired recycling of the membrane-associated proteins. Another recent NGS study involving 213 PD patients revealed three novel *VPS35* variations (i.e., p.P316S, p.Y507F, and p.E787K), leading to changes in coded amino acids potentially contributing to PD pathogenesis. Additionally, a specific mutation in the eukaryotic translation initiation factor 4 gamma 1 (*EIF4G1*) (p.R1205H) was identified as a robust PD risk factor in the same study [17]. Nonetheless, a significant proportion of inherited PD cases remain genetically unexplained.

Compared to typical adult-geriatric disease, early-onset PD is well suited for NGS-based studies given its greater chances to be associated with rare multiple variants. Using WES and homozygosity mapping, Edvardson et al. detected a deleterious mutation in the DnaJ heat shock protein family (Hsp40) member C6 (*DNAJC6*) (c.801-2A>G) in two subjects affected by juvenile parkinsonism. This mutation has been associated with abnormal transcripts and a marked reduction in *DNAJC6* mRNA levels [18]. Coincidentally, by mapping the disease locus with a lod score of 5.13 to a <3.5 Mbp region at 1p31.3 in a consanguineous family and through a subsequent WES analysis, Köroğlu et al. [19] identified a homozygous truncating mutation (p.Q734X) in the *DNAJC6* gene. These findings further confirm the role of *DNAJC6* as a gene associated with juvenile parkinsonism, expanding the spectrum of parkinsonism phenotypes and *DNAJC6* mutations [19].

Based on these considerations, in this study, we used NGS techniques to identify new variants potentially associated with PD, as well as to assess which already known variants are present, in a homogeneous cohort of Sicilian subjects with PD.

## 2. Materials and Methods

### 2.1. Participants

The study included a convenience sample of 126 PD patients (84 males and 42 females) with a mean age of 73.18 years (standard deviation: 10.88 years) and an average disease duration of 6.06 ± 4.63 years, all diagnosed according to the latest diagnostic criteria for PD [20]. All the participants were Caucasian and of Sicilian ancestry, and were recruited from the Oasi Research Institute—IRCCS of Troina (EN), Italy.

Additional details regarding the clinical–demographic characteristics of these PD patients, along with their primary comorbidities and medication(s) taken, are provided in the Supplementary Table S1. Notably, 20 patients had a positive family history for PD. Among all patients, 62 exhibited an akinetic-rigid phenotype, 21 presented with a tremor-dominant phenotype, and the remaining 43 displayed mixed features. Furthermore, 43 patients had clinical and video-polysomnography evidence of RBD, while other sleep disorders (including insomnia, obstructive sleep apnea syndrome, and periodic limb movement during sleep, with or without concurrent RBD) were detected in 91 subjects. In terms of cognitive status, 31 patients had very mild or mild cognitive impairment, while 23 exhibited various degrees of severity of dementia. Seventeen subjects were diagnosed with a depressive disorder, with some experiencing concomitant anxiety. Additionally, 55 patients had neuroimaging evidence of chronic subcortical vascular disease, and most patients (90) presented one or more conventional vascular risk factor, with hypertension being the most prevalent. At the time of examination, 55 patients were drug-naive, while the others were being treated with one or more anti-parkinsonian drugs (30 were on levodopa alone, 29 were on levodopa + other drugs, and 12 were on anti-parkinsonian drugs other than levodopa).

Informed consent for study participation was obtained from all enrolled patients or, if needed, from their relatives. The Ethics Committee of the Oasi Research Institute—IRCCS of Troina (Italy) approved the protocol on 5 April 2022 (approval code: 2022/04/05/CE-IRCCS-OASI/52) and the study was carried out according to the Declaration of Helsinki in 1964 and its later amendments.

### 2.2. DNA Extraction

DNA extraction was initiated from peripheral blood samples collected in EDTA tubes. We followed the protocol by Lahiri and Nurnberger [21], which is a cost-effective, safe, and efficient method for preparing DNA from whole blood.

### 2.3. NGS Sequencing

NGS experiments, including sample quality control, were performed by Genomix4life S.R.L. of Baronissi (Italy). The DNA concentration was assessed using a NanoDropOne spectrophotometer (Thermo Fisher Scientific, Waltham, MA, USA), and the quality was evaluated with an TapeStation 4200 (Agilent Technologies, Santa Clara, CA, USA). Indexed libraries were created from 300 ng/µL of purified DNA using DNA Prep with Enrichment with TruSight One Panel (Illumina, San Diego, CA, USA), which provides comprehensive coverage of over 4800 disease-associated genes. Library quantification was performed using the Agilent TapeStation 4200 and Qubit fluorometer (Thermo Fisher Scientific, Waltham, MA, USA), and the libraries were subsequently pooled to ensure equimolar amounts of each index-tagged sample, resulting in a final sample concentration of 2 nM. Sequencing and cluster generation were performed with the Illumina NextSeq550Dx system in a 2 × 150 paired-end format, with ~100× coverage. The sequence files (.fastq files) were subjected to a quality control analysis through the FastQC (http://www.bioinformatics.babraham.ac.uk/projects/fastqc/ (accessed on 17 October 2023). Paired-end reads were aligned to the NCBI reference sequence (GRCh37/hg19) and alignment and variant calling were performed in BaseSpace using Burrows-Wheeler Aligner and Genome Analysis Toolkit (Burrows-Wheeler Aligner enrichment application).

An NGS panel of 162 genes, selected for their known associations in the literature with degenerative movement disorders, was applied (Supplementary Table S2).

The raw data are available at ArrayExpress (E-MTAB-13523), accessed on 14 October 2023.

### 2.4. Data Analysis and Annotation

Identified variants were filtered based on allele frequencies (mean frequency, MAF) < 1%, utilizing the 1000 Genomes and ExAC as reference genomic datasets. In silico analyses were conducted using data obtained from wANNOVAR, with input files provided in .vcf format. Each variant was associated with the clinical profile and supported by literature references from The Human Gene Mutation Database (HGMD). Variants classified as “Disease Causing mutation (DM)” or “Disease-Causing mutation? (DM?)” were selected from the analysis results.

### 2.5. Statistical Analysis

A statistical analysis was employed to compare the PD patients with variants classified as “DM” and “DM?”. Specifically, the frequencies of these variants in males/females and their association with cognitive features were assessed using the Chi-Square test, with a significance threshold set at *p* < 0.05.

## 3. Results

Applying the filters described in the “Data analysis and annotation” section, we found that 76 subjects did not have detected variants. Conversely, 50 samples (30 from males and 20 from females) yielded positive results for the studied gene panel, resulting in the identification of a total of 44 variants (with some subjects having multiple variants). Among these 44 variants (see Table 1), 26 (as documented in the HGMD and related literature) are associated with PD, while the remaining 18 are linked to other movement or neuromuscular disorders. Notably, for 8 of the 162 genes included in the panel, i.e., *GBA*, *HTRA2*, microtubule-associated protein tau (*MAPT*), *LRRK2*, DNA Polymerase Gamma (*POLG*), *PRKN*, senataxin (*SETX*), and tenascin R (*TNR*), 2 or more variants were identified (Table 1). On the contrary, the other 18 genes exhibited only one variant each (Table 1).

**Figure 1:**
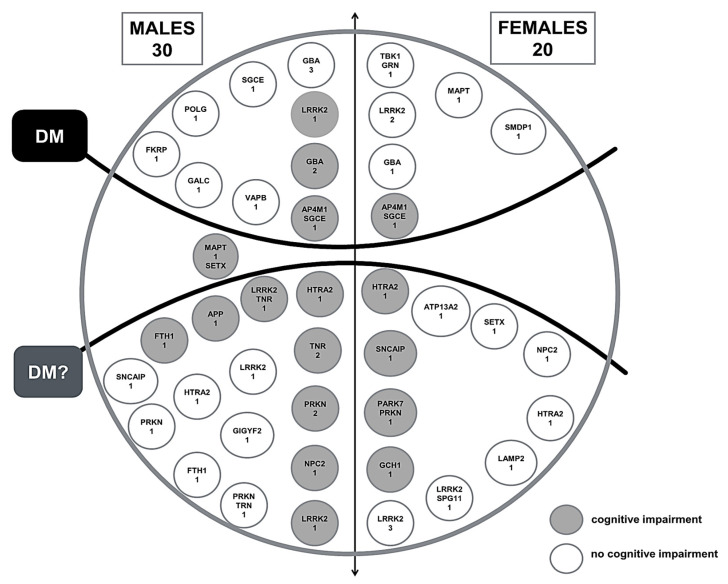
Scheme differentiating patients listed in Table 1 by sex, “DM” and “DM?”, and the presence/absence of cognitive impairment. Each dot also reports the genes and the number of patients in whom it was found. Please see the text for gene abbreviations.

The genes with the highest number of identified variants were *LRRK2*, *GBA*, and *PRKN*, with 9, 4, and 3 variants, respectively. In total, we identified 19 “DM” variants and 25 “DM?” variants (Table 1). The statistical analysis of the two groups of patients with “DM” or “DM?” variants revealed no significant differences regarding either sex or the presence/absence of cognitive impairment (Figure 1).

## 4. Discussion

The main finding emerging from this study is that 60% of the clinically diagnosed PD samples (76 out of 126) did not exhibit any variant in the genes we studied, thus suggesting that other genes may be involved. At the same time, the present study confirms the multifactorial pathophysiology of PD, where the genetic component is not always necessarily dominant in the development of the disease, and the role played by the complex interaction between environmental factors and genetic susceptibility [65,66].

In the review by Karimi-Moghadam et al. (2018) and by Day and Mullin (2021) [65,66], all major genes implicated in genetic subtypes, familial monogenic forms, and sporadic forms were listed. These included *SNCA*, *PARKIN*, *UCHL1*, *PINK1*, *DJ-1*, *LRRK2*, *ATP13A2*, *GIGYF2*, *HTRA2*, *PLA2G6*, *VPS35*, *EIF4G1*, *DNAJC6*, synaptojanin 1 (*SYNJ1*), DnaJ heat shock protein family (Hsp40) member C13 (*DNAJC13*), coiled-coil-helix-coiled-coil-helix domain containing 2 (*CHCHD2*), vacuolar protein sorting 13 homolog C (*VPS13C*), *GBA*, spinocerebellar ataxia 2 (*SCA2*), transmembrane protein 230 (*TMEM230*), dynactin subunit 1 (*DCTN1*), and *POLG*. However, despite several Whole-Genome Association Studies conducted on PD, heterogeneous results have been produced regarding the occurrence of genetic variants in these patients [65,66]. It is worth mentioning that all the aforementioned genes belong to the panel studied in this research using NGS, as shown in Table 1. Globally, we observed variants in the *LRRK2*, *ATP13A2*, *GIGYF2*, *GBA*, *HTRA2*, and *POLG* genes, which are all associated with PD.

**Figure 2:**
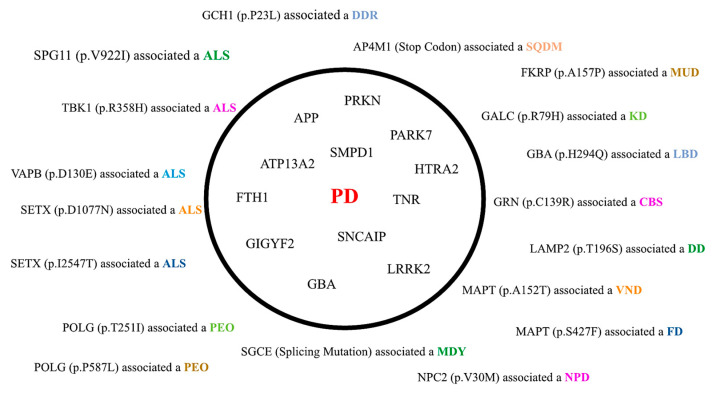
Diagram highlighting all variants and genes not associated with Parkinson’s disease but with other neurodegenerative or neuromuscular disorders (located outside the central circle), while PD-associated genes are represented within the circle (please also refer to Table S2 for additional details). Please see the legend of Table 1 for abbreviations.

Additionally (Table 1; Figure 2), we identified a variant of the *SNCA* gene, which was associated with PD as “DM?”. For the *GBA* gene, we identified four variants: three have been already associated with PD [28,29,30], while the other with Lewy-body dementia [31]; of note, all these variants were identified as “DM.” Regarding the *LRRK2* gene, we identified three variants as “DM” associated with PD [38,41,46], whereas six variants were detected as “DM?” associated with PD [39,40,42,43,44,45]. Also, for the *HTRA2*, *GIGYF2*, and *ATP13A2* genes, we identified variants detected as “DM?” and associated with PD [24,33,35,36]. As shown in Table 1, *PARKIN*, *PRKN* alternative title, was found with three variants [53,54,55] associated with PD as “DM?”. Notably, our results showed two variants on the *POLG* gene, both in the same patient, which the literature has currently associated with progressive external ophthalmoplegia (PEO) [51,52]. However, this patient did not exhibit any clinical manifestation of PEO, thus suggesting that even variants in genes related to mitochondrial activity might play an important role in PD pathogenesis [67].

All the other genes and variants listed in Table 1 and Figure 2 are not currently associated with PD, but with other movement disorders. Interestingly, five variants in four different genes have been reported in ALS (Table 1; Figure 2), although in the present study, they were clinically associated with PD. These genes are the following:

- *SPG11*, which encodes for spatacsin, a protein with a role in neuronal axonal growth, function, and intracellular trafficking [68];
- *TBK1*, required for efficient recruitment in autophagy; mutations in the *TBK1* gene may result in impaired autophagy and contribute to the accumulation of protein aggregates in ALS [69];
- *VAPB*, encoding for a protein that is part of the vesicle-associated membrane protein family, plays a role in suppressing the accumulation of unfolded proteins within the endoplasmic reticulum [70];
- *SETX*, an ATP-dependent helicase required for unwinding and resolution of RNA:DNA hybrids (R-loops) formed during transcription [71].

All of these genes are involved in protein transportation and, when mutated, can lead to protein accumulation within neuronal cells, a crucial step commonly observed in several neurodegenerative diseases [72,73,74,75]. Therefore, both protein accumulation and the lack of adequate protein clearance play a key role in neurodegeneration. Translating to our study, we cannot exclude the possibility that genes related to protein transport may be involved not only in ALS (as previously described), but also in PD and other degenerative movement disorders [76,77,78]. Moreover, it can be hypothesized that variants associated with clinical phenotypes other than PD might a;sp play a role in PD development as genetic cofactors. This would reinforce the concept that PD is, at least in the majority of cases, a multigenic and multifactorial disorder within a complex environmental context.

Lastly, cognitive impairment is a relevant non-motor manifestation of PD. In the present study, no significant difference was found regarding the association between cognitive impairment and sex, although a trend towards an association with male sex was noted (Figure 1): 5 out of 20 females (25%) and 12 out of 30 males (40%) had cognitive impairment. Therefore, a higher risk of cognitive impairment in males with PD might be hypothesized, although this was evaluated only among the variants in the genes we studied, thus warranting further evidence. The association with cognitive impairment occurred only in case of concomitant presence of variants in the *AP4M1* gene (c.1117C>T; stop codon) and the *SGCE* gene (c.232+1G>T; splicing mutation) (Table 1), which were detected in one male and one female patient. As such, the simultaneous occurrence of these two variants seems to confer a higher predisposition for cognitive impairment.

This study has limitations. First, the cohort size was relatively small, although it was clinically homogeneous and was carefully screened and selected. Second, we used a panel of genes rather than sequencing the entire exome; this imposes constraints on the ability to identify new susceptibility genes, although it enhances the depth of sequencing for the selected genes. Nevertheless, this study may pave the way towards more unbiased approaches using the whole-genome sequencing contributing to the knowledge of the multifactorial and/or environmental character of PD.

## 5. Conclusions

The results obtained for the *LRRK2*, *ATP13A2*, *GIGYF2*, *GBA*, *HTRA2*, and *POLG* genes confirm the literature and underscore that some PD-causing mutations are universally significant. Simultaneously, novel gene variants, presently associated with other movement disorders or neurodegenerative diseases, appear to be linked to PD. Among these genes, mutations in *POLG* underscore the role of mitochondrial alterations in PD, along with the clinical and research significance of the five variants previously associated with ALS. Moreover, but no less important, cognitive impairment appears to be more closely associated with males in PD. A deeper understanding of the genetic basis of PD, coupled with the identification of new variants potentially linked to the disease, will enhance diagnostic accuracy, broaden therapeutic applications, and refine prognostic implications for affected individuals.

## Figures and Tables

**Table 1:** List of all the genetic variants, filtered according to HGMD, and the association with PD or other degenerative movement disorders according to the literature.

ID	Sex	Gene	NM	Variant	HGDM	Reference	Condition
9PD 3PD	F M	*AP4Ml*	NM_004722.4	c.1117C>T; STOP CODON; Et.	DM	[22]	SQDM
134PD	M	*APP*	NM_000484	c.1795G>A (p.E599K); MISSENSE; Et.	DM?	[23]	PD
79PD	F	*ATP13A2*	NM_022089	c.2836A>T (p.1946F); MISSENSE; Et.	DM?	[24]	PD
102PD	M	*FKRP*	NM_024301.5	c.469G>C (p.Al57P); MISSENSE; Om.	DM	[25]	MUD
44PD 55PD	M M	*FTHl*	NM_002032	c.161A>G (p.K54R); MISSENSE	DM?	[26]	PD
108PD	M	*GALC*	NM_000153.4	c.236G>A (p.R79H); MISSENSE; Et.	DM	[27]	KD
128PD 57PD	M M	*GBA*	NM_001005741	c.1226A>G (p.N409S); MISSENSE; Et.	DM	[28]	PD
98PD 116PD	M F	*GBA*	NM_001005741	c.1448T>C (p.L483P); MISSENSE; Et.	DM	[29]	PD
130PD	M	*GBA*	NM_001005741	c.1223C>T (p.T408M); MISSENSE; Et.	DM	[30]	PD
llPD	M	*GBA*	NM_001005741	c.882T>G (p.H294Q); MISSENSE; Et.	DM	[31]	LBD
132PD	F	*GCHl*	NM_000161	c.68C>T (p.P23L); MISSENSE; Et.	DM?	[32]	DDR
87PD	M	*GIGYF2*	NM_001103146	c.1370A>C (p.N457T); MISSENSE; Et.	DM?	[33]	PD
106PD	F	*GRN*	NM_002087.4	c.415T>C (p.C139R); MISSENSE; Et.	DM	[34]	CBS
54PD	F	*HTRA2*	NM_013247	c.215T>C (p.L72P); MISSENSE; Et.	DM?	[35]	PD
101PD 121PD 137PD	M F M	*HTRA2*	NM_013247	c.1195G>A (p.G399S); MISSENSE; Et.	DM?	[36]	PD
123PD	F	*LAMP2*	NM_002294	c.586A>T (p.T196S); MISSENSE; Et.	DM?	[37]	DD
107PD	F	*LRRK2*	NM_198578.4	c.6055G>A (p.G2019S); MISSENSE; Et.	DM	[38]	PD
5PD 63PD	M F	*LRRK2*	NM_198578	c.4541G>A (p.R1514Q); MISSENSE	DM?	[39]	PD
89PD	F	*LRRK2*	NM_198578	c.5467C>A (p.Q1823K); MISSENSE; Et.	DM?	[40]	PD
117PD	F	*LRRK2*	NM_198578	c.lOOOG>A (p.E334K); MISSENSE; Et.	DM	[41]	PD
30PD	F	*LRRK2*	NM_198578	c.356T>C (p.L119P); MISSENSE; Et.	DM?	[42]	PD
4PD	M	*LRRK2*	NM_198578	c.6929C>T (p.T2310M); MISSENSE; Et.	DM?	[43]	PD
29PD	F	*LRRK2*	NM_198578	c.7067C>T (p.T23561); MISSENSE; Et.	DM?	[44]	PD
31PD	M	*LRRK2*	NM_198578	c.3200G>A (p.R1067Q); MISSENSE; Et.	DM?	[45]	PD
32PD	M	*LRRK2*	NM_198578	c.6566A>G (p.Y2189C); MISSENSE; Et.	DM	[46]	PD
59PD	M	*MAPT*	NM_005910	c.454G>A (p.A152T); MISSENSE; Et.	DM	[47]	VND
67PD	F	*MAPT*	NM_016835	c.1280C>T (p.S427F); MISSENSE; Et.	DM	[48]	FD
2PD 74PD	M F	*NPC2*	NM_006432	c.88G>A (p.V30M); MISSENSE; Et.	DM?	[49]	NPD
94PD	F	*PARK7*	NM_007262	c.293G>A (p.R98Q); MISSENSE; Et.	DM?	[50]	PD
48PD	M	*POLG*	NM_002693	c.1760C>T (p.P587L); MISSENSE; Et.	DM	[51]	PEO
48PD	M	*POLG*	NM_002693	c.752C>T (p.T2511);	DM	[52]	PEO
lOOPD 45PD 78PD	M M M	*PRKN*	NM_004562	c.1204C>T (p.R402C); MISSENSE; Et.	DM?	[53]	PD
94PD	F	*PRKN*	NM_004562	c.245C>A (p.A82E); MISSENSE; Et.	DM?	[54]	PD
68PD	M	*PRKN*	NM_004562	c.lOOOC>T (p.R334C); MISSENSE;	DM?	[55]	PD
46PD	F	*SETX*	NM_015046	c.7640T>C (p.12547T); MISSENSE; Et.	DM?	[56]	ALS
59PD	M	*SETX*	NM_015046	c.3229G>A (p.D1077N); MISSENSE; Et.	DM?	[57]	ALS
9PD 3PD 104PD	F M M	*SGCE*	NM_003919.3	c.232+1G>T; SPLICING MUTATION; Et.	DM	[58]	MDY
lPD	F	*SMPDl*	NM_000543	c.1550A>C (p.E517V); MISSENSE; Et.	DM	[59]	PD
88PD 37PD	F M	*SNCAIP*	NM_005460	c.2125G>C (p.E709Q); MISSENSE	DM?	[60]	PD
63PD	F	*SPGll*	NM_025137	c. 2764G>A (p.V9221); MISSENSE	DM?	[61]	ALS
106PD	F	*TBKl*	NM_013254.4	c.1073G>A (p.R358H); MISSENSE; Et.	DM	[62]	ALS
115PD 5PD	M M	*TNR*	NM_003285	c.496A>G (p.T166A); MISSENSE; Et.	DM?	[63]	PD
135PD 68PD	M M	*TNR*	NM_003285	c.538A>C (p.N180H); MISSENSE; Et.	DM?	[63]	PD
97PD	M	*VAPB*	NM_004738	c.390C>T (p.D130E); MISSENSE; Et.	DM	[64]	ALS

Legend: NM_ identifies the reference sequence of a transcript; HGMD—Human Gene Mutation Database; Et.—heterozygosity; Om.—homozygosity; SQDM—spastic-dystonic quadriplegia, delayed myelination; MUD—muscular dystrophy; KD—Krabbe’s disease; LBD—Lewy body dementia; DDR—dystonia dopa-responsive; CBS—corticobasal syndrome; DD—Danon’s disease; VND—various neurodegenerative diseases; FD—Frontotemporal dementia; NPD—Niemann–Pick’s disease, type C2; PEO—progressive external ophthalmoplegia; ALS—amyotrophic lateral sclerosis; MDY—myoclonus dystonia; AP4M1—adaptor-related protein complex 4 subunit mu 1; APP—amyloid precursor protein; FKRP—fukutin related protein; FTH1—ferritin heavy chain 1; GALC—galactosylceramidase; GCH1—GTP cyclohydrolase 1; GRN—granulin precursor; LAMP2—lysosomal associated membrane protein 2; NPC2—NPC intracellular cholesterol transporter 2; SGCE—sarcoglycan epsilon; SMPD1—sphingomyelin phosphodiesterase 1; SNCAIP—synuclein alpha interacting protein; SPG11—SPG11 vesicle trafficking associated, spatacsin; TBK1—TANK binding kinase 1; VAPB—VAMP associated protein B and C; REF.—Reference; PAT. ASSOC. REF—pathology-associated reference; M—male; and F—female.

## Data Availability

The raw data are available at ArrayExpress (E-MTAB-13523), accessed on 14 October 2023.

## Supplementary Materials

The following supporting information can be downloaded at: https://www.mdpi.com/article/10.3390/biomedicines11123118/s1, Table S1: clinical-demographic characteristics of PD patients enrolled, along with their main comorbidities and medication(s) taken; Table S2: the table lists all 162 genes in the NGS gene panel used to analyze all subjects.
