# Supplementary material for: A Next-Generation Sequencing Study in a Cohort of Sicilian Patients with Parkinson’s Disease
Source: Biomedicines. 2023 Nov 22;11(12):3118. doi: 10.3390/biomedicines11123118 (PMC10740523; doi:10.3390/biomedicines11123118)
Supplement: Supplementary file 1 [file biomedicines-11-03118-s001.zip › Supplementary Table S2.pdf]

Supplementary Table S2. The table lists the 162 genes within the NGS gene panel, which was used to analyze all the subject participants.

**Gene Panel**	*ABCA7, ABCB1, ACMSD, ACVR2B, ADH1C, ADORA1, ALS2, ANG, ANKRD13A, APEX1, APOE, APP, ASAH1, ASCL1, ATG12, ATG5, ATG7, ATP13A2, ATP1A3, ATP6P2, ATXN2, ATXN3, BDNF, C9ORF72, CDKN1A, CHCHD2, CHRNB3, COL12A1, COMT, COQ2, CP, CPXM1, CR1, CSMD1, CTSD, CYP2D6, DBH, DCTN1, DNAJC12, DNAJC13, DNAJC6, DNM1L, DRD4, DYNC1H1, EEF1D, EIF4G1, EPPK1, ERBB2, FBXL17, FBXO7, FGF20, FMR1, FTH1, FTL, FUS, GAK, GBA, GCH1, GIGYF2, GLA, GLUD2, GPA, GRN, GSK3B, HLADRA, HMOX1, HMOX2, HNMT, HSPA1A, HSPA9, HTRA2, IL1B, KCNV2, LAMP2, LCT, LRRK1, LRRK2, MAPT, MGA, MKS1, MTHFR, MTX1, NDUFV2, NEFM, NME8, NOD2, NPC1, NPC2, NQO2, NR4A2, PACRG, PARK2, PARK7, PARL, PEPD, PGK1, PINK1, PITX3, PLA2G6, PLXNA4, PMEL, PODXL, POLG, PRKN, PRNP, PRNP, PSEN2, PTEN, PTK2B, PTPRH, RAB29, RAB39B, RAD51B, RIC3, RUNDC3A, SCARB2, SCN9A, SETX, SIRT1, SLC17A5, SLC18A2, SLC30A10, SLC39A14, SLC41A1, SLC52A1, SLC5A9, SLC6A3, SLC6A4, SLURP1, SMPD1, SNCA, SNCAIP, SNCB, SOD1, SOD2, SORL1, SPG11, SPR, SYNJ1, TAF1, TARDBP, TBP, TENT2, TH, TMEM230, TNK2, TNR, TRAP1, TREM2, UCHL1, UHRF1, USP24, VAC14, VAPB, VCP, VPS13C, VPS35, VPS53, WARS2, XRCC1, XRCC3, ZFYVE26*
